# Identification of key candidate genes and biological pathways in bladder cancer

**DOI:** 10.7717/peerj.6036

**Published:** 2018-12-04

**Authors:** Xin Gao, Yinyi Chen, Mei Chen, Shunlan Wang, Xiaohong Wen, Shufang Zhang

**Affiliations:** Central Laboratory, Central South University Xiangya School of Medicine Affiliated Haikou Hospital, Haikou, China

**Keywords:** Bladder cancer, GEO database, Integrated bioinformatics, Differentially expressed genes, Biological pathways

## Abstract

**Background:**

Bladder cancer is a malignant tumor in the urinary system with high mortality and recurrence rates. However, the causes and recurrence mechanism of bladder cancer are not fully understood. In this study, we used integrated bioinformatics to screen for key genes associated with the development of bladder cancer and reveal their potential molecular mechanisms.

**Methods:**

The GSE7476, GSE13507, GSE37815 and GSE65635 expression profiles were downloaded from the Gene Expression Omnibus database, and these datasets contain 304 tissue samples, including 81 normal bladder tissue samples and 223 bladder cancer samples. The RobustRankAggreg (RRA) method was utilized to integrate and analyze the four datasets to obtain integrated differentially expressed genes (DEGs), and the gene ontology (GO) functional annotation and Kyoto encyclopedia of genes and genomes (KEGG) pathway analysis were performed. Protein-protein interaction (PPI) network and module analyses were performed using Cytoscape software. The OncoLnc online tool was utilized to analyze the relationship between the expression of hub genes and the prognosis of bladder cancer.

**Results:**

In total, 343 DEGs, including 111 upregulated and 232 downregulated genes, were identified from the four datasets. GO analysis showed that the upregulated genes were mainly involved in mitotic nuclear division, the spindle and protein binding. The downregulated genes were mainly involved in cell adhesion, extracellular exosomes and calcium ion binding. The top five enriched pathways obtained in the KEGG pathway analysis were focal adhesion (FA), PI3K-Akt signaling pathway, proteoglycans in cancer, extracellular matrix (ECM)-receptor interaction and vascular smooth muscle contraction. The top 10 hub genes identified from the PPI network were vascular endothelial growth factor A (VEGFA), TOP2A, CCNB1, Cell division cycle 20 (CDC20), aurora kinase B, ACTA2, Aurora kinase A, UBE2C, CEP55 and CCNB2. Survival analysis revealed that the expression levels of ACTA2, CCNB1, CDC20 and VEGFA were related to the prognosis of patients with bladder cancer. In addition, a KEGG pathway analysis of the top 2 modules identified from the PPI network revealed that Module 1 mainly involved the cell cycle and oocyte meiosis, while the analysis in Module 2 mainly involved the complement and coagulation cascades, vascular smooth muscle contraction and FA.

**Conclusions:**

This study identified key genes and pathways in bladder cancer, which will improve our understanding of the molecular mechanisms underlying the development and progression of bladder cancer. These key genes might be potential therapeutic targets and biomarkers for the treatment of bladder cancer.

## Introduction

Bladder cancer, which is a common malignant tumor in the genitourinary system, has high mortality and recurrence rates, and poses a serious threat to people’s lives and health. The incidence of bladder cancer is ranked 7th in men and 17th in women (Burger et al., 2013). The gold standard for the diagnosis of bladder cancer is cystoscopy and urine cytology. Cystoscopy is invasive, complicated to perform and expensive, and makes patients feel uncomfortable; urine cytology involves the use of exfoliated cells in excreted urine to diagnose bladder cancer and has a low sensitivity for low-grade tumors. The ideal method for the detection of bladder cancer should be more convenient and rapid; thus, biomarkers of bladder cancer have become a research focus in recent years. The recurrence of bladder cancer recurrence has always been a major challenge in the treatment of this type of cancer, although patients can be effectively treated or even cured after routine postoperative bladder infusion chemotherapy, the recurrence rate remains 60% (Liu et al., 2015). At present, approximately 70–80% of bladder tumors initially diagnosed in the clinic are non-muscle invasive bladder cancer (NMIBC) (Alfred et al., 2017). For patients with NMIBC, transurethral resection of the bladder tumor (TURBT) is considered the preferred surgical option; however, 45–80% of patients with NMIBC may relapse and progress (Clark et al., 2016; Kukreja & Shah, 2017). NMIBC often progresses to muscle invasive bladder cancer (MIBC) after recurrence, and MIBC is associated with in a poor prognosis. Therefore, in-depth studies of the potential molecular mechanisms underlying the malignant biological behavior of bladder cancer cells would aid the identification of reliable molecular markers for the early diagnosis, prognosis evaluation and recurrence monitoring, and the development of methods for controlling the proliferation of bladder cancer cells and the exploration of new drug targets are important.

Gene chips, also known as DNA microarrays, are a type of biochip and have become an important means for obtaining information about cancer gene expression profiles in a large-scale and highly efficient manner (Lu & Zhang, 2006). The gene expression omnibus (GEO) database is a large and comprehensive public gene expression data resource that contains a variety of tumor gene expression profile datasets (Jiang & Liu, 2015). Microarray analysis is a novel method used for researching tumor genes, searching for molecular targets of tumor drug therapies and monitoring prognosis. However, due to the heterogeneity of experimental samples, the use of different detection platforms and data processing methods will lead to inconsistent results. The RobustRankAggreg (RRA) method is suitable for comparing several sequenced gene lists (Kolde et al., 2012), because this method reviews the ranking of each gene in each list and is based on the assumption that each gene identified in each experiment is randomly arranged. Thus, RRA compares the ranking of a randomly ordered list with the baseline case, and a higher gene rank is associated with a lower *P*-value. The integration of the results of multiple gene expression datasets by RRA provide a better understanding of the molecular mechanisms of tumor genes (Liu et al., 2018).

In this study, we downloaded four original gene chip expression profile datasets, namely, GSE7476, GSE13507, GSE37815 and GSE65635, from the GEO database, and these datasets contain a total of 304 samples, including 81 normal bladder tissue samples and 223 bladder cancer samples. We used a Perl language command to convert the chip probe ID to a gene symbol and then used R language software to standardize all the datasets. The RRA method was subsequently used to integrate the results and obtain integrated differentially expressed genes (DEGs), and the obtained DEGs were analyzed using the DAVID database for the gene ontology (GO) functional annotation and kyoto encyclopedia of genes and genomes (KEGG) pathway analysis. A protein-protein interaction (PPI) network of the DEGs was constructed using the search tool for the retrieval of interacting genes/proteins (STRING) database; then, hub genes were identified, and modules were constructed from the PPI network. The OncoLnc online tool was utilized to analyze the relationships between hub genes and patient prognosis. Our research will provide reliable molecular markers and effective therapeutic targets for bladder cancer.

## Materials and Methods

### Microarray data

The GSE7476, GSE13507, GSE37815 and GSE65635 gene expression profile matrix files were downloaded from the GEO database (https://www.ncbi.nlm.nih.gov/geo/). The platform of the GSE7476 dataset is the GPL570 [HG-U133_Plus_2] Affymetrix Human Genome U133 Plus 2.0 Array, and this dataset contains three normal bladder tissues and nine bladder cancer tissues. The GSE13507, the platform of which is the GPL6102 Illumina human-6 v2.0 expression beadchip, contains 68 normal bladder tissues and 188 bladder cancer tissues. The GSE37815, the platform of which is the GPL6102 Illumina human-6 v2.0 expression beadchip, contains six normal bladder tissues and 18 bladder cancer tissues. The platform of the GSE65635 dataset is the GPL14951 Illumina HumanHT-12 WG-DASL V4.0 R2 expression beadchip, and it contains four normal bladder tissues and eight bladder cancer tissues. The series matrix TXT files and platform TXT files were downloaded. The dataset information is shown in Table 1, and the clinical information of the samples in each dataset is shown in Table S1. A Perl language command was used to convert the gene probe IDs in the matrix files to the gene symbols in the platform files to obtain a matrix file containing the international standard gene name. Each dataset was then normalized using the normalize Between Arrays function in the limma R package (http://www.bioconductor.org/). All gene expression data were subjected to log2 transformation.

**Table 1:** Details of the GEO bladder cancer data.

Reference	Sample	GEO	Platform	Normal	Tumor
Mengual et al. (2009)	bladder	GSE7476	GPL570	3	9
Kim et al. (2010), Lee et al. (2010)	bladder	GSE13507	GPL6102	68	188
Kim et al. (2013)	bladder	GSE37815	GPL6102	6	18
M. V. Suntsova (2015, unpublished data)	bladder	GSE65635	GPL14951	4	8

**Note:**

GEO, gene expression omnibus.

### Screening for DEGs

The limma R package was used to screen for DEGs in each dataset. Genes with a corrected *P*-value < 0.05 and |log fold change (FC)| > 1 were considered DEGs. The upregulated and downregulated gene lists were saved as Excel files, and the TXT files of all gene lists sorted by logFC in each dataset were saved for subsequent integration analysis.

### Integration of microarray data

The four TXT files of all gene lists sorted by logFC were integrated using the RobustRankAggreg (RRA) R package (https://cran.rstudio.com/bin/windows/contrib/3.5/RobustRankAggreg_1.1.zip), and the integrated upregulated and downregulated DEG lists were saved for subsequent analysis.

### GO and KEGG pathway enrichment analyses of DEGs

The DAVID 6.8 database (https://david.ncifcrf.gov/) is a commonly used database for gene enrichment and functional annotation analyses. This database integrates biological data and analytical tools to provide systematic and comprehensive annotation of biological functions for large-scale lists of genes or proteins. The GO annotation and KEGG pathway enrichment analyses of the identified DEGs were performed using DAVID, and the TXT files of the results of the GO and KEGG pathway enrichment analyses were downloaded for subsequent analysis. A visual network analysis of the KEGG analysis results was performed using Cytoscape 3.6.1 software. The results were considered statistically significant if *P* < 0.05.

### PPI network construction and analysis of modules

Search Tool for the Retrieval of Interacting Genes/Proteins is a search tool that can analyze the interaction relationships between proteins (https://string-db.org/). The use of STRING to analyze the PPI network of DEGs can help us understand the relationships between different genes. Cytoscape software was used to screen for hub genes according to degrees (Song et al., 2018). The modules in the PPI network were analyzed using the MCODE plug-in in Cytoscape software with the default parameters “Degree Cutoff = 2,” “Node Score Cutoff = 0.2,” “K-Core = 2” and “Max.Depth = 100” (Bader & Hogue, 2003).

### Prognosis analysis

The prognosis associated with the hub genes was analyzed using the OncoLnc online analysis tool (http://www.oncolnc.org/), which combines prognostic data from The Cancer Genome Atlas (TCGA) database with mRNA, miRNA or lncRNA expression levels. The percentiles of the low expression group and the high expression group were set to 50%. The expression and prognosis data for each gene were downloaded, and Kaplan–Meier curves were drawn using online tools.

## Results

### Microarray data normalization and identification of DEGs in bladder cancer

**Figure 1:**
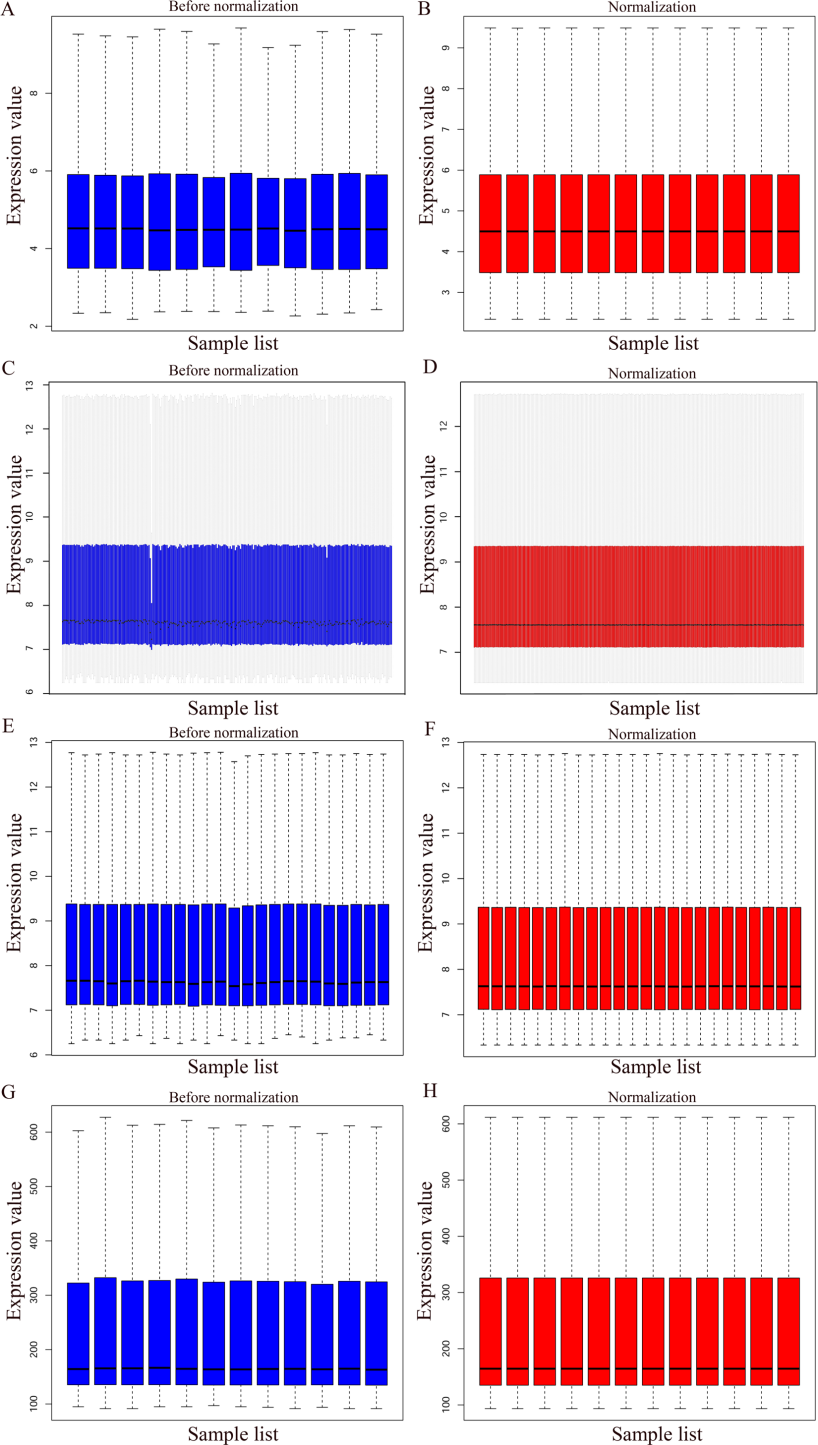
Normalization of gene expression. (A–B) Normalization of the GSE7476 data set. (C–D) Normalization of the GSE3785 data set. (E–F) Normalization of the GSE37815 data set. (G–H) Normalization of the GSE65635 data set. Blue represents data before normalization, and red represents data after normalization.

**Figure 2:**
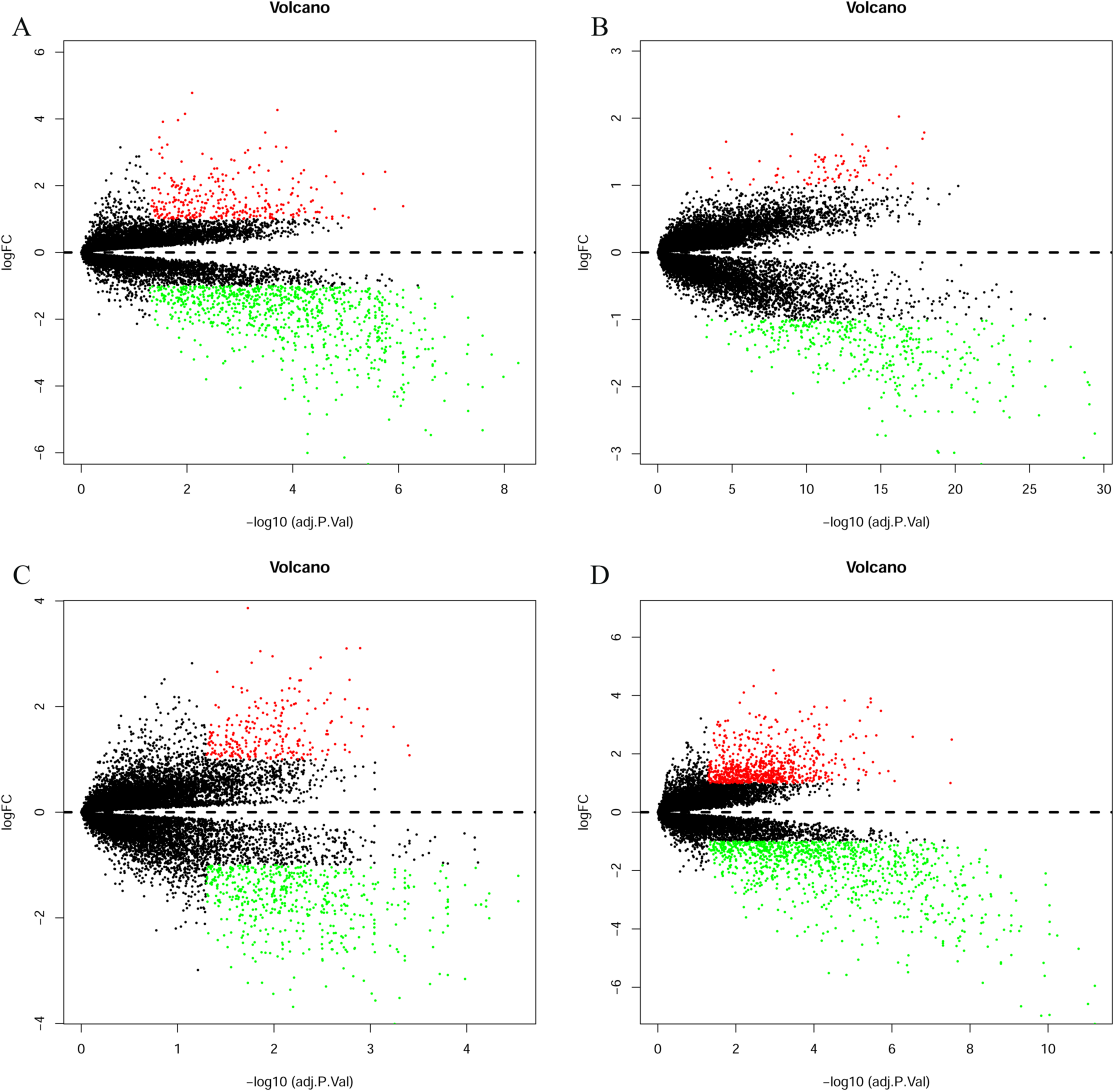
Differentially expressed genes between the two groups of samples in each dataset. (A) GSE7476, (B) GSE13507, (C) GSE37815, (D) GSE65635. The red dots represent the upregulated genes based on an adjusted *P* < 0.05 and |log fold change| > 1; the green dots represent the downregulated genes based on an adjusted *P* < 0.05 and |log fold change| > 1; the black spots represent genes with no significant difference in expression.

**Figure 3:**
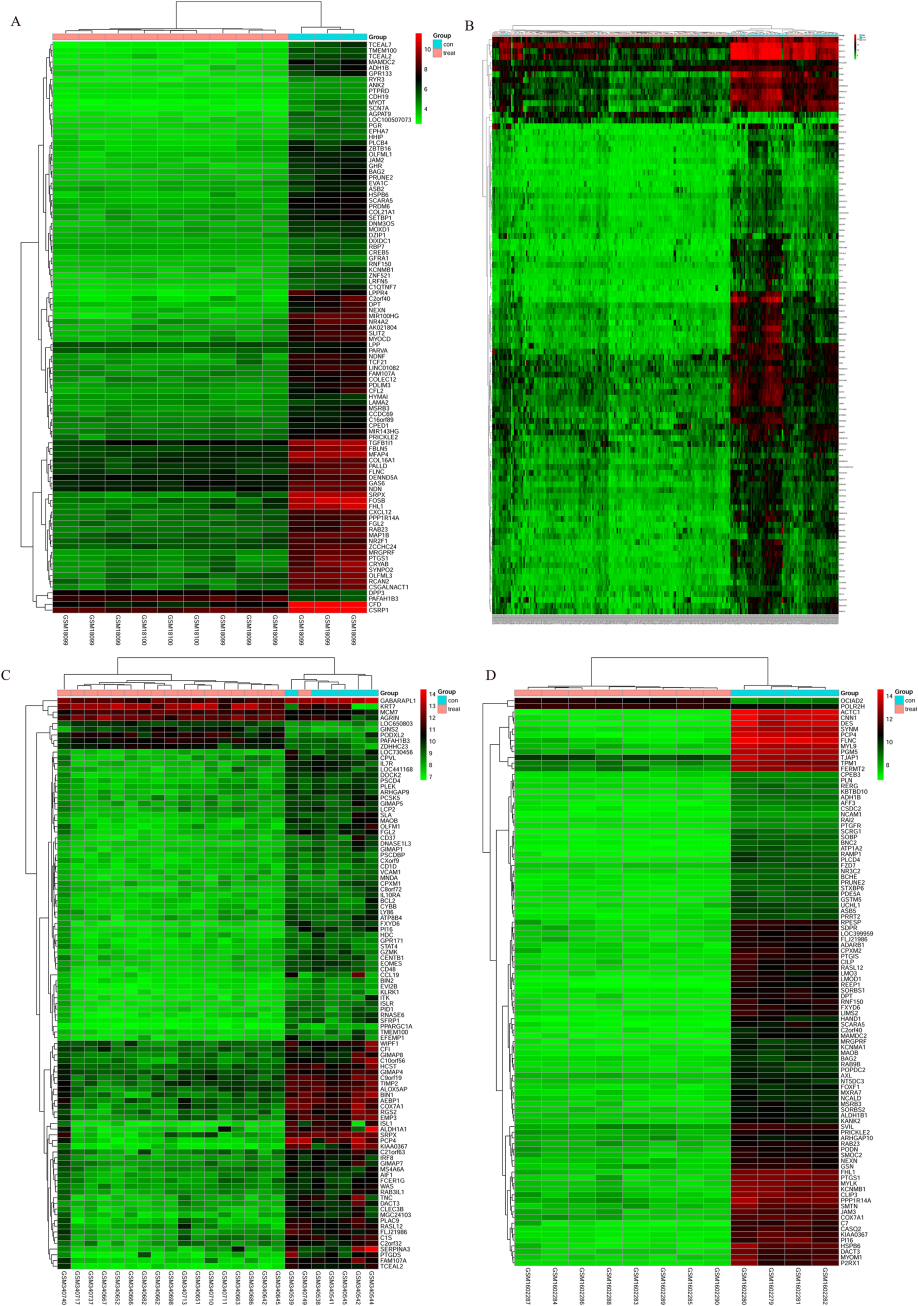
Cluster heat map of the top 100 DEGs. (A) GSE7476, (B) GSE13507, (C) GSE37815, (D) GSE65635. Red indicates relative upregulation of gene expression; green indicates the relative downregulation of gene expression; black indicates no significant change in gene expression; and gray indicates that the signal intensity is not high enough to detect.

The bladder cancer chip expression datasets GSE7476, GSE13507, GSE37815 and GSE65635 were normalized, and the results are shown in Fig. 1. The DEGs were screened using the limma R package (adjusted *P* < 0.05 and |log fold change (FC)| > 1). The GSE7476 dataset contained 1,174 differential genes, including 318 upregulated genes and 856 downregulated genes. The GSE13507 dataset contained 464 differential genes, including 77 upregulated genes and 388 downregulated genes. The GSE37815 dataset contained 780 differential genes, including 247 downregulated expression genes and 533 downregulated expression genes. In addition, the GSE65635 dataset contained 1,759 differential genes, including 773 upregulated genes and 986 downregulated genes. The DEGs of the four datasets are shown in Fig. 2, and the cluster heat map of the top 100 genes is shown in Fig. 3.

### Identification of integrated DEGs in bladder cancer using integrated bioinformatics

**Figure 4:**
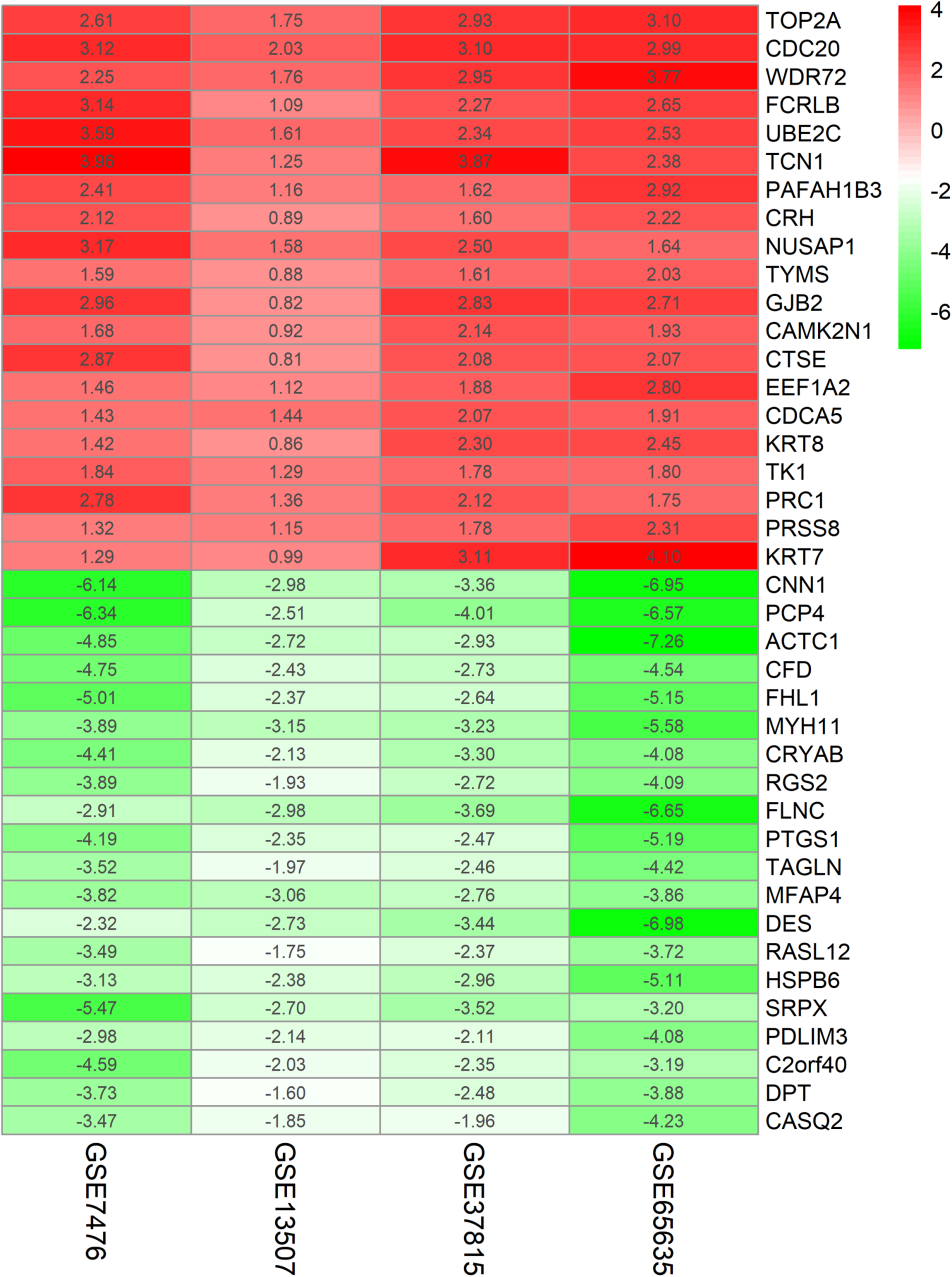
Log FC heatmap of each expression microarray. The abscissa represent the GEO IDs, the ordinate represents the gene name, the red represents log FC > 0, the green represents log FC < 0 and the value in the box represents the log FC value.

The chip expression matrix of the four datasets was obtained using the limma R package and sorted according to logFC. The integrated DEGs were screened using the RRA package (corrected *P* < 0.05, logFC > 1 or −logFC < −1). The RRA method is based on the assumption that each gene in each dataset is randomly arranged. If the gene ranks high in all datasets, the associated *P*-value is lower, the possibility of differential gene expression is greater. Through rank analysis, 343 integrated DEGs, consisting of 111 upregulated genes and 232 downregulated genes, were identified by the RRA method (Table S2). The top 20 upregulated genes and the top 20 downregulated genes were mapped on heat map, as shown in Fig. 4.

### Functional enrichment analysis of DEGs

**Figure 5:**
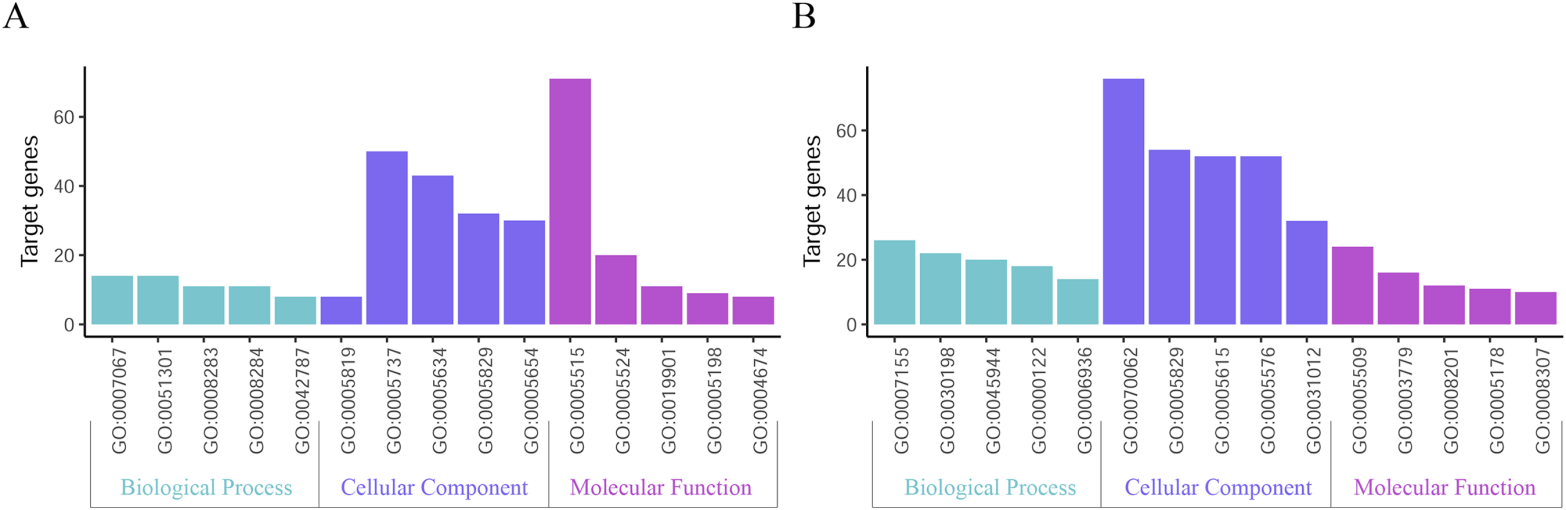
Top 15 enriched GO terms. (A) Upregulated DEGs with the top 15 enriched GO terms. (B) Downregulated DEGs with the top 15 enriched GO terms.

**Figure 6:**
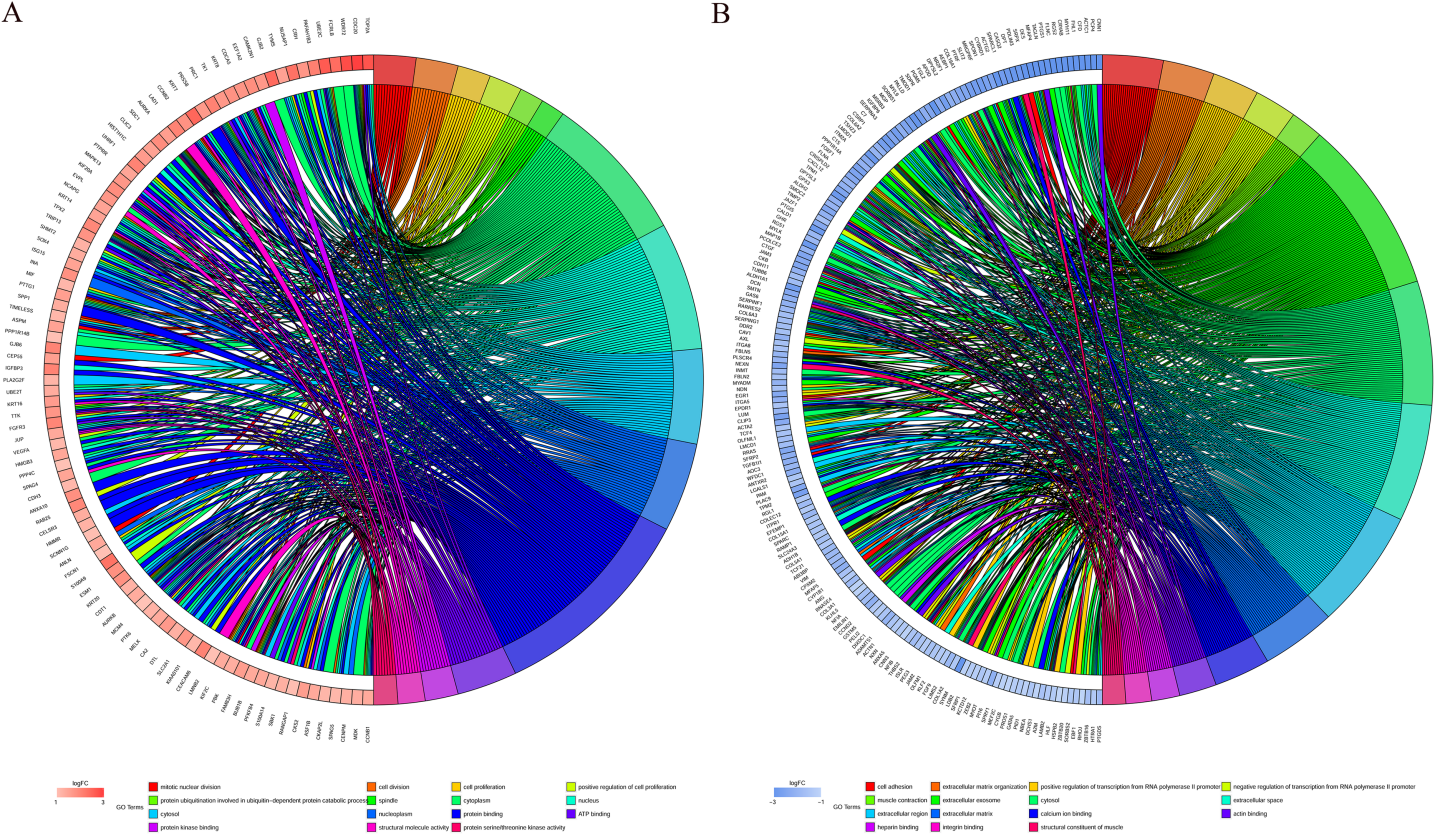
Distribution of integrated DEGs in bladder cancer for different GO-enriched functions. (A) Upregulated DEGs. (B) Downregulated DEGs.

GO function annotation of the integrated DEGs was performed using the DAVID database and its online analysis tool. The GO functional analysis of the integrated differential genes was divided into the following three parts: biological process (BP), molecular function (MF) and cell component (CC). The results were considered statistically significant if *P* < 0.05, and the three parts of the GO results are shown in Figs. 5 and 6. The top 15 results obtained from the GO enrichment analysis of the upregulated and downregulated differential genes are shown in Table 2. The upregulated genes were mainly enriched in mitotic nuclear division (ontology: BP), the spindle (ontology: CC), and protein binding (ontology: MF) and the downregulated genes were mainly enriched in cell adhesion (ontology: BP), extracellular exosomes (ontology: CC) and calcium ion binding (ontology: MF).

**Table 2:** Top 15 GO enrichment terms associated with the upregulated and downregulated genes.

A. Upregulated genes top 15 enriched GO terms.			
Category	Term	Count	*P*-value
BP	mitotic nuclear division	14	2.64E-09
BP	cell division	14	1.56E-07
BP	cell proliferation	11	6.49E-05
BP	positive regulation of cell proliferation	11	4.58E-04
BP	protein ubiquitination involved in ubiquitin-dependent protein catabolic process	8	3.58E-05
CC	spindle	8	6.76E-06
CC	cytoplasm	50	8.86E-05
CC	nucleus	43	0.018272
CC	cytosol	32	0.003596
CC	nucleoplasm	30	9.69E-04
MF	protein binding	71	1.15E-04
MF	ATP binding	20	9.88E-04
MF	protein kinase binding	11	7.14E-05
MF	structural molecule activity	9	1.02E-04
MF	protein serine/threonine kinase activity	8	0.006637

B. Downregulated genes top 15 enriched GO terms.			
Category	Term	Count	*P*-value
BP	cell adhesion	26	9.09E-10
BP	extracellular matrix organization	22	6.93E-14
BP	positive regulation of transcription from RNA polymerase II promoter	20	0.04382
BP	negative regulation of transcription from RNA polymerase II promoter	18	0.010419
BP	muscle contraction	14	9.69E-10
CC	extracellular exosome	76	1.22E-11
CC	cytosol	54	0.022837
CC	extracellular space	52	2.83E-13
CC	extracellular region	52	2.08E-10
CC	extracellular matrix	32	3.93E-20
MF	calcium ion binding	24	2.90E-05
MF	actin binding	16	2.03E-06
MF	heparin binding	12	4.99E-06
MF	integrin binding	11	7.08E-07
MF	structural constituent of muscle	10	1.80E-09

**Notes:**

BP, biological process.

CC, cellular component.

MF, molecular function.

### KEGG pathway analysis of DEGs

**Figure 7:**
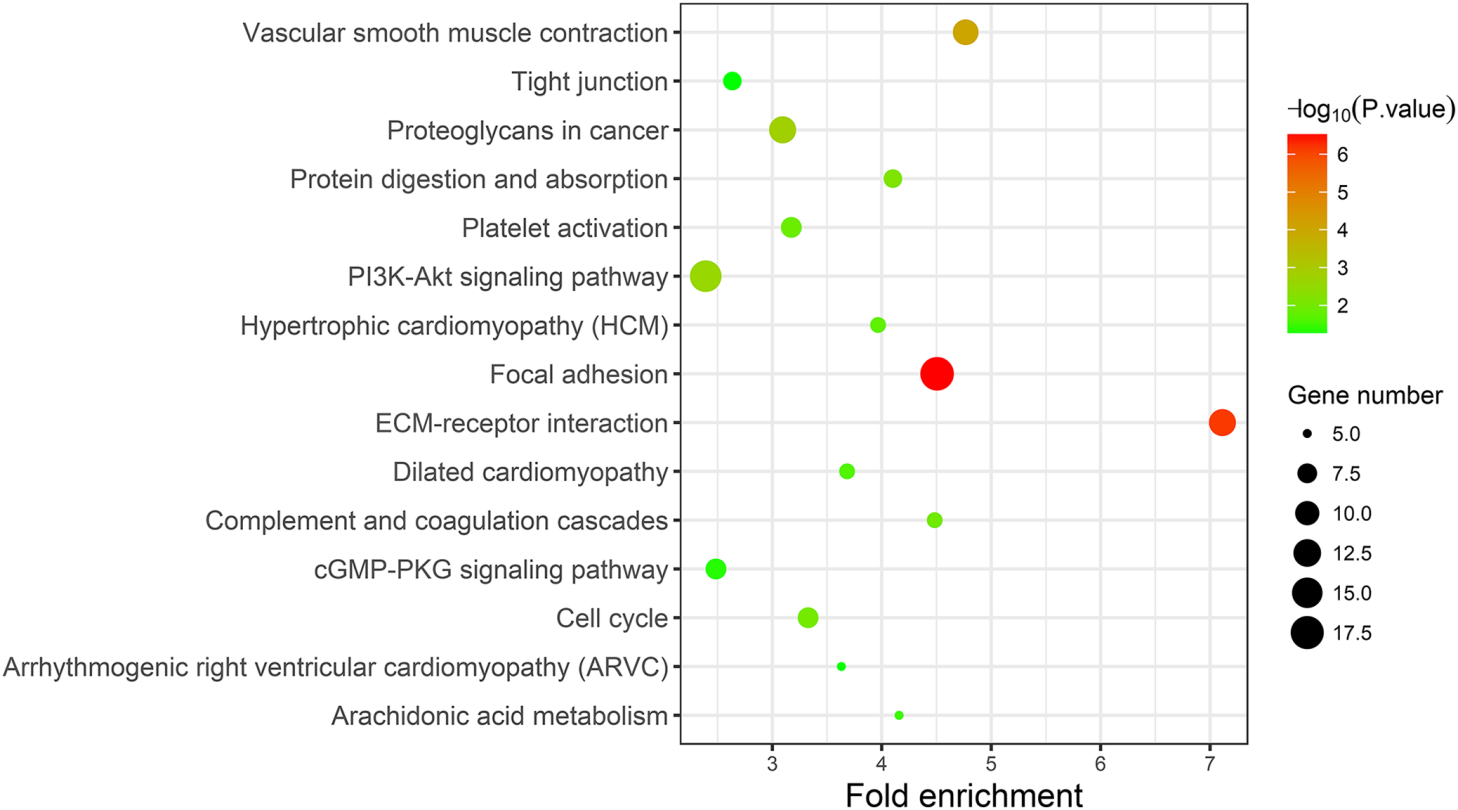
KEGG pathway enrichment analysis of the integrated DEGs.

**Figure 8:**
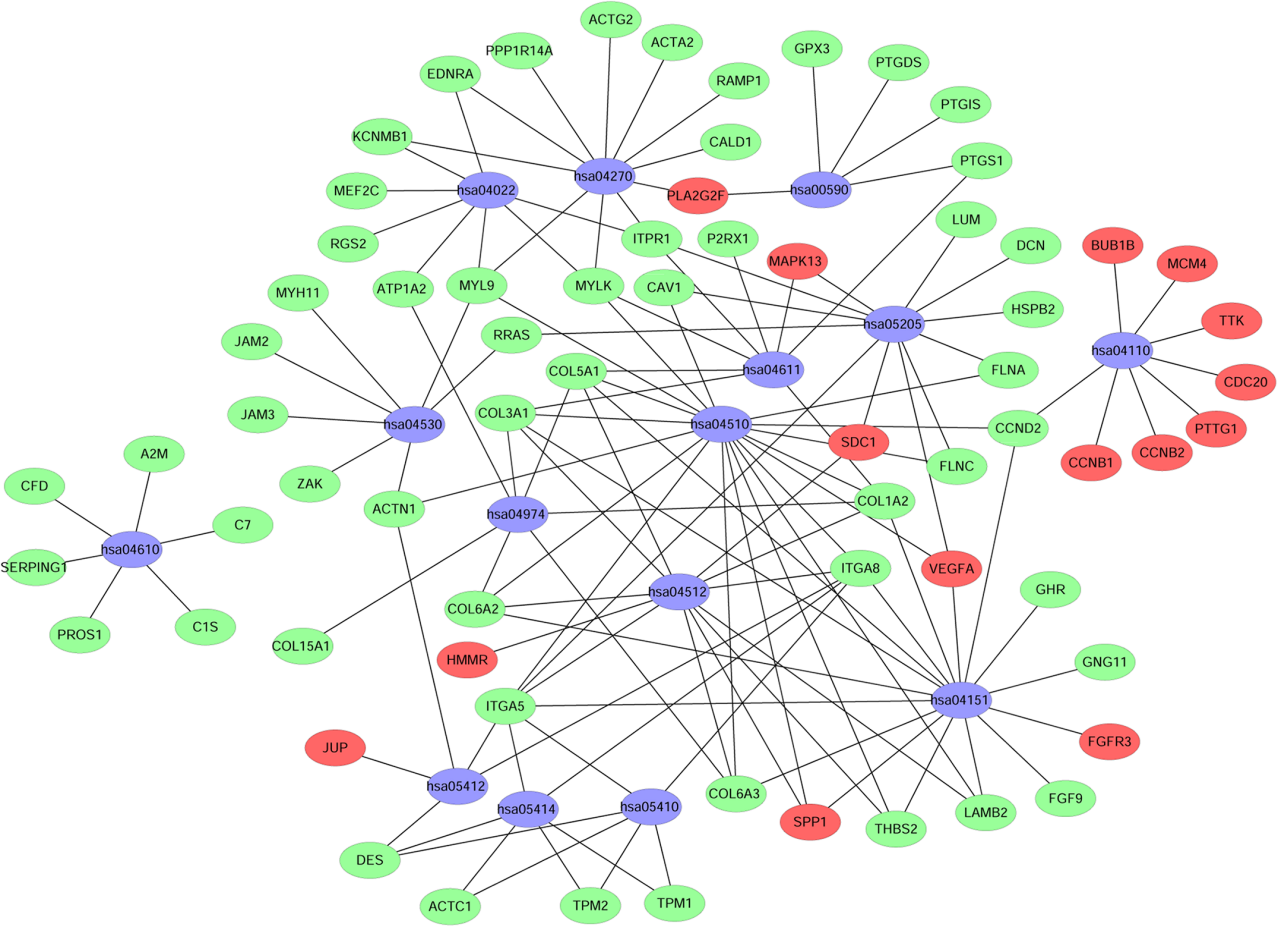
Network map of enriched pathways. Blue represents the pathways, red represents the upregulated genes and green represents the downregulated genes.

A KEGG pathway analysis of the integrated DEGs was performed using the DAVID database, and the results of the analysis are shown in Table 3 and Fig. 7. The integrated DEGs were mainly enriched in five pathways, namely, focal adhesion (FA), the PI3K-Akt signaling pathway, proteoglycans in cancer, extracellular matrix (ECM)-receptor interaction, and vascular smooth muscle contraction. The network diagram was drawn using Cytoscape software, and the results are shown in Fig. 8.

**Table 3:** Kyoto Encyclopedia of Genes and Genomes (KEGG) pathway analysis of integrated DEGs.

Pathway	ID	Gene count	*P*-value	Genes
Focal adhesion	hsa04510	18	3.50E-07	*CAV1 COL3A1 ACTN1 FLNC FLNA COL5A1 MYL9 LAMB2 ITGA5 CCND2 ITGA8 COL6A3 VEGFA COL1A2 COL6A2 THBS2 MYLK SPP1*
ECM-receptor interaction	hsa04512	12	7.16E-07	*SDC1 LAMB2 ITGA5 ITGA8 COL3A1 COL6A3 COL1A2 COL6A2 THBS2 COL5A1 SPP1 HMMR*
Vascular smooth muscle contraction	hsa04270	11	8.92E-05	*EDNRA ACTG2 ACTA2 CALD1 RAMP1 MYLK ITPR1 KCNMB1 PPP1R14A PLA2G2F MYL9*
Proteoglycans in cancer	hsa05205	12	0.001565	*CAV1 SDC1 MAPK13 ITGA5 LUM VEGFA HSPB2 RRAS DCN FLNC FLNA ITPR1*
PI3K-Akt signaling pathway	hsa04151	16	0.002477	*FGFR3 FGF9 COL3A1 GNG11 COL5A1 LAMB2 ITGA5 CCND2 ITGA8 VEGFA COL6A3 COL6A2 COL1A2 THBS2 GHR SPP1*
Protein digestion and absorption	hsa04974	7	0.006777	*COL3A1 COL6A3 COL1A2 COL6A2 COL15A1 ATP1A2 COL5A1*
Cell cycle	hsa04110	8	0.009743	*CCNB1 CCNB2 CCND2 TTK BUB1B CDC20 PTTG1 MCM4*
Complement and coagulation cascades	hsa04610	6	0.01029	*C7 A2M SERPING1 C1S CFD PROS1*
Platelet activation	hsa04611	8	0.012441	*P2RX1 MAPK13 COL3A1 PTGS1 COL1A2 MYLK ITPR1 COL5A1*
Hypertrophic cardiomyopathy (HCM)	hsa05410	6	0.016864	*ACTC1 DES ITGA5 ITGA8 TPM2 TPM1*
Dilated cardiomyopathy	hsa05414	6	0.022525	*ACTC1 DES ITGA5 ITGA8 TPM2 TPM1*
Arachidonic acid metabolism	hsa00590	5	0.031006	*PTGIS PTGDS GPX3 PTGS1 PLA2G2F*
cGMP-PKG signaling pathway	hsa04022	8	0.040657	*EDNRA MEF2C RGS2 ATP1A2 MYLK ITPR1 KCNMB1 MYL9*
Arrhythmogenic right ventricular cardiomyopathy (ARVC)	hsa05412	5	0.047397	*JUP DES ITGA5 ITGA8 ACTN1*
Tight junction	hsa04530	7	0.048279	*ZAK MYH11 RRAS ACTN1 JAM2 JAM3 MYL9*

### Integration of protein-protein interaction (PPI) network and module analysis

The STRING online database was used to analyze the 343 integrated DEGs and to construct a PPI network. The results were downloaded and analyzed using Cytoscape software. The top 10 hub genes were screened according to their degree values, and the identified hub genes were vascular endothelial growth factor A (VEGFA), TOP2A, CCNB1, Cell division cycle 20 (CDC20), aurora kinase B (AURKB), ACTA2, Aurora kinase A (AURKA), UBE2C, CEP55 and CCNB2, as shown in Table 4.

**Table 4:** The degree values of the top 10 hub genes.

Gene symbol	Gene description	LogFC	Degree
*VEGFA*	Vascular endothelial growth factor A	1.17	57
*TOP2A*	Topoisomerase (DNA) II alpha	2.60	52
*CCNB1*	Cyclin B1	1.40	44
*CDC20*	Cell division cycle 20	2.81	42
*AURKB*	Aurora kinase B	1.36	41
*ACTA2*	Actin, alpha 2, smooth muscle, aorta	−2.36	41
*AURKA*	Aurora kinase A	1.58	39
*UBE2C*	Ubiquitin-conjugating enzyme E2C	2.52	39
*CEP55*	Centrosomal protein 55	1.56	39
*CCNB2*	Cyclin B2	1.77	38

**Note:**

FC, fold change.

**Figure 9:**
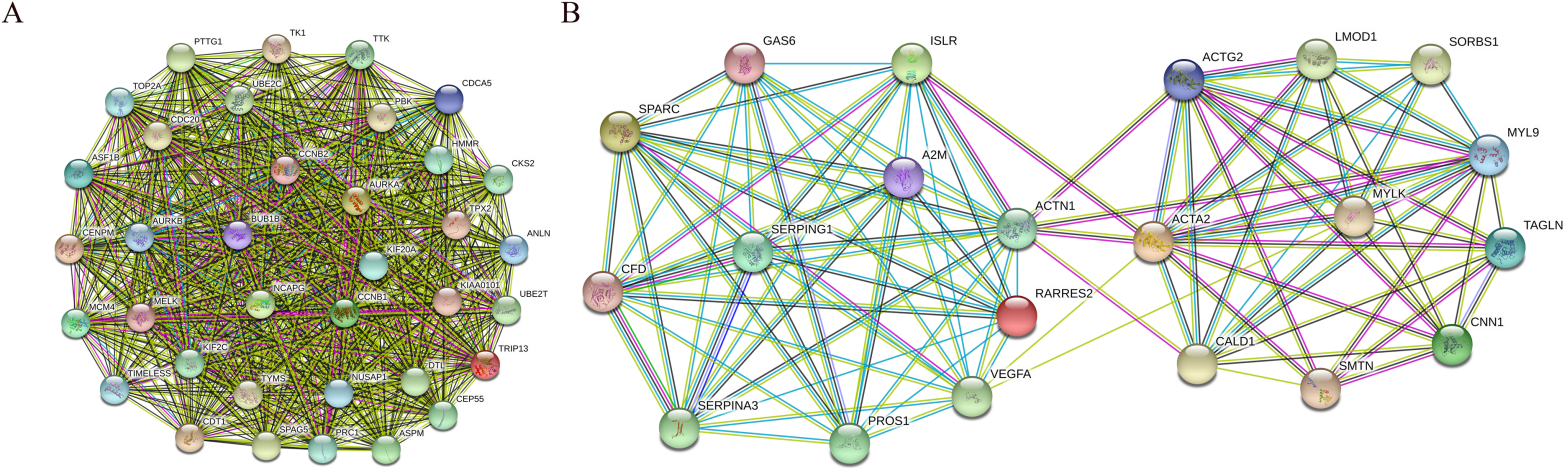
PPI network of module 1 (A) and module 2 (B). Circles represent genes, lines represent interactions between gene-encoded proteins and line colors represent evidence of interactions between proteins.

In addition, nine functional modules were screened from the PPI network using MCODE, and the two most important modules were selected, as shown in Fig. 9. A KEGG pathway enrichment analysis of all the genes in both modules was performed using the DAVID database (Table 5). The results showed that the genes in Module 1 are mainly enriched in the cell cycle and oocyte meiosis, and the genes in Module 2 are mainly enriched in the complement and coagulation cascades, vascular smooth muscle contraction and FA.

**Table 5:** KEGG enrichment of genes in the top 2 modules.

Module	Pathway	Count	*P*-value	Genes
Module1	Cell cycle	7	8.18E-08	*CCNB1, CCNB2, BUB1B, TTK, CDC20, PTTG1, MCM4*
	Oocyte meiosis	5	6.09E-05	*CCNB1, CCNB2, CDC20, AURKA, PTTG1*
Module2	Complement and coagulation cascades	4	3.18E-04	*A2M, SERPING1, CFD, PROS1*
	Vascular smooth muscle contraction	4	0.001033	*ACTG2, ACTA2, CALD1, MYLK, MYL9*
	Focal adhesion	4	0.004874	*VEGFA, ACTN1, MYLK, MYL9*

### Prognostic significance of hub genes in patients with bladder cancer

**Figure 10:**
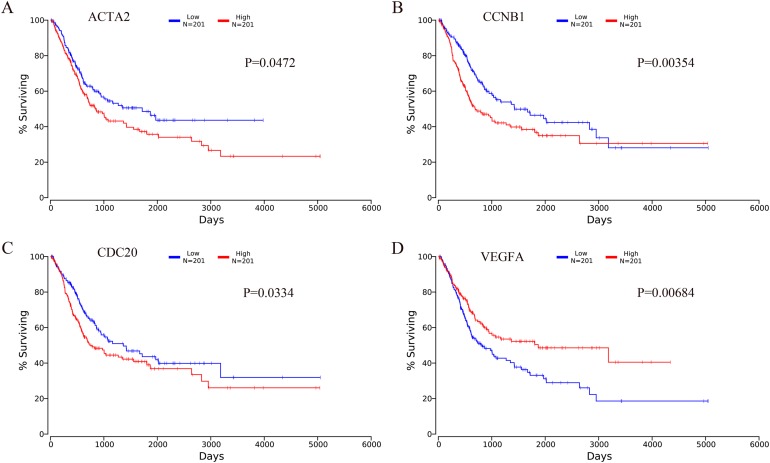
Kaplan–Meier analysis results of hub genes (*P* < 0.05). Four hub genes were found to be associated with the prognosis of bladder cancer patients. (A) ACTA2; (B) CCNB1; (C) CDC20; (D) VEGFA.

The OncoLnc online analysis tool was used to analyze the prognosis of bladder cancer according to the expression of the top 10 hub genes. The analysis tool can perform Kaplan–Meier prognosis analyses based on gene expression and the prognostic correlation in the TCGA database. Among the 10 hub genes, the following four genes were found to be associated with the prognosis of bladder cancer patients: ACTA2 (*P* = 0.0472), CCNB1 (*P* = 0.00354), CDC20 (*P* = 0.0334) and VEGFA (*P* = 0.00684). The Kaplan–Meier analysis results are shown in Fig. 10.

## Discussion

Bladder cancer is a common malignant tumor in the urogenital system. The incidence of bladder cancer ranks 11th among malignant tumors, and approximately 150,000 people die of bladder cancer each year (Siegel, Miller & Ahmedin Jemal, 2015). At present, transurethral resection of bladder tumors is the most common operation for noninvasive bladder cancer, but the recurrence rate is high. In fact, the recurrence rates in high-risk bladder cancer patients are 50% in one year and 90% in five years (Lightfoot et al., 2014). The occurrence of bladder cancer is characterized by its multicentered nature, and the mechanisms underlying its occurrence and development are complex. Therefore, researching the mechanisms underlying bladder cancer development is very important.

In this study, the GSE7476, GSE13507, GSE37815 and GSE65635 datasets were analyzed using the RRA method, and 343 integrated DEGs were found. The 343 integrated DEGs were then subjected to BP, CC and MF enrichment analysis. The upregulated genes were mainly enriched in mitotic nuclear division (ontology: BP), the spindle (ontology: CC), and protein binding (ontology: MF), and the downregulated genes were mainly enriched in cell adhesion (ontology: BP), extracellular exosomes (ontology: CC) and calcium ion binding (ontology: MF). These results suggest that these DEGs are involved in the proliferation and migration of bladder cancer cells. The KEGG pathway analysis revealed that these integrated DEGs are mainly enriched in the following top five pathways: FA, the PI3K-Akt signaling pathway, proteoglycans in cancer, ECM-receptor interaction and vascular smooth muscle contraction. FA is a membrane-related macromolecule assembly that links the actin cytoskeleton and integrin to the extracellular matrix. It plays an important role in the maintenance of cell tension and signal transduction for cell survival. In recent years, numerous studies have shown that FA-related structural molecules are involved in the regulation of tumor cell epithelial mesenchymal transition (EMT) processes and the promotion of tumor invasion and metastasis (Akrida et al., 2018; Desgrosellier et al., 2014; Li et al., 2018a; Nam et al., 2012; Yang et al., 2018a). Focal adhesion kinases (FAK) can be involved in carcinogenic signaling in the invasion and migration of bladder cancer cells (Kong, Chen & Sima, 2017). Neoh et al. (2017) reported that inhibition of the expression of MMP-2 and MMP-9 regulated by the FAK/PI3K/AKT/mTOR pathway can inhibit bladder cancer cell migration and invasion. The phosphatidylinositol 3-kinase/protein kinase B (PI3K/Akt) signaling pathway plays an important role in the occurrence and development of tumors. Overactivation of the PI3K/Akt signaling pathway promotes the malignant transformation of cells by regulating tumor cell proliferation, apoptosis, migration, invasion, angiogenesis, immune evasion and drug resistance (De Santis et al., 2017; Mulholland et al., 2012; Ruiz-Saenz et al., 2018; Soumya et al., 2013; Yang et al., 2018b), whereas inhibition of the PI3K/Akt signaling pathway can inhibit the growth cycle of bladder cancer cells (Li et al., 2018b). The ECM is one of the most abundant components in the tumor microenvironment. Collagen is closely related to the function of the ECM in influencing the biological behavior of tumor cells (Riegler et al., 2018). The functions of epithelial cells, including cell differentiation, migration and invasion, are guided by physical interaction with the ECM (Lu, Weaver & Werb, 2012). Changes in collagen type I in the ECM microenvironment might promote the progression of non-myometrial invasive bladder cancer (Brooks et al., 2016). Therefore, studying these pathways would help elucidate the mechanisms underlying the proliferation and invasion of bladder cancer and aid the prediction of cancer progression.

We also constructed PPI networks with 343 integrated DEGs and identified the following 10 hub genes: VEGFA, TOP2A, CCNB1, CDC20, AURKB, ACTA2, AURKA, UBE2C, CEP55 and CCNB2. We performed a prognostic analysis of those 10 hub genes using the OncoLnc online analysis tool. The results showed that the expression levels of ACTA2, CCNB1, CDC20 and VEGFA were associated with the prognosis of patients with bladder cancer. VEGFA can promote angiogenesis (Pauty et al., 2018). VEGFA is also believed to directly increase the metastatic potential of cancer cells (Ghosh et al., 2008). Zaravinos et al. (2012) found that VEGFA is highly expressed in bladder cancer, and Pignot et al. (2009) found that stage T3-T4 bladder cancers with VEGFA overexpression are the most likely to benefit from antiangiogenic therapy. VEGFA is not only a promising therapeutic target but also a good prognostic factor for MIBC. Topoisomerase (DNA) II alpha (TOP2A) encodes a DNA topoisomerase, an enzyme that controls and alters the topologic states of DNA during transcription, and high expression levels of TOP2A are significantly associated with shorter survival rates in cancer patients (Ren et al., 2018). Kim et al. (2010) found that enhanced expression of TOP2A is positively correlated with the high recurrence and progression rates of NMIBC, and TOP2A might be a prognostic marker for NMIBC. However, further research is needed to elucidate the exact mechanism of TOP2A in the development and progression of bladder cancer. Both CCNB1 and CCNB2 belong to the cyclin (CCN) family, and numerous studies have shown that CCNB1 is overexpressed and promotes tumor proliferation in a variety of tumors, such as breast cancer, colorectal cancer and hepatocellular carcinomas (Chai et al., 2018; Fang et al., 2014; Li et al., 2013). Kim et al. (2014) found that the recurrence of NMIBC might be mediated by FOXM1-CCNB1-Fanconi anemia pathways, and is significantly related to the expression of CCNB1. CCNB2 is also highly expressed in a variety of tumors, such as colorectal adenocarcinoma, breast cancer and bladder cancer (Lei et al., 2016; Park et al., 2007; Shubbar et al., 2013). In addition, another study showed that CCNB2 overexpression is associated with clinical progression and poor prognosis in non-small-cell lung cancer (Qian et al., 2015). CDC20 is generally considered to be a carcinogenic factor that promotes tumor development (Kidokoro et al., 2008; Wang et al., 2013). Consistent with our study, increased expression of CDC20 in patients with bladder cancer has been associated with poor prognosis (Choi et al., 2013). AURKA and AURKB in the aurora kinase family are closely related to the occurrence and development of malignant tumors. AURKA is a cell cycle-associated serine-threonine kinase that is overexpressed in various types of cancer and is strongly associated with poor prognosis (Marampon et al., 2014). Mobley et al. (2017) found that the knock down AURKA has little effect on the proliferation of bladder cancer cells but prevents the invasion of tumor cells and that the overexpression of AURKA was associated with poor prognosis. AURKB is a key regulator of malignant mitosis and is involved in chromosome segregation and cytokinesis. Bufo et al. (2010) found that high expression of AURKB might be involved in the development of bladder cancer and hypothesized that bladder cancer could be treated by targeting AURKB expression with specific anti-mitotic agents in the future. ACTA2 contributes to the maintenance of the mechanical tension and shape of cells and is also crucial for tumor cell migration and invasion (Lambrechts, Van & Ampe, 2004). Lee et al. (2013) found that lung adenocarcinoma patients with high expression levels of ACTA2 have significantly increased distant metastasis and poor prognosis. Wu et al. (2017) conducted mass spectrometry and bioinformatics analyses and found that ACTA2 might play an important role in the progression of bladder cancer, which is consistent with our study, but the specific regulatory mechanisms and prognosis associated with ACTA2 in bladder cancer remain not fully understood. The ubiquitin-conjugating enzyme E2C (UBE2C) plays a key role in the regulation of mitotic processes. A previous study showed that UBE2C expression is significantly associated with the progression of myometrial invasive bladder cancer (Fristrup et al., 2013). Morikawa et al. (2013) found that UBE2C positivity is significantly associated with a higher tumor stage and lymphangiogenesis, which suggests that UBE2C might be a new prognostic biomarker and therapeutic target for bladder cancer. The main functions of centrosomal protein 55 (CEP55) are to anchor microtubule-associated proteins, participate in spindle formation, and regulate cell proliferation. CEP55 overexpression is significantly associated with tumor stage, invasiveness, metastasis and poor prognosis of multiple tumor types (Jeffery et al., 2016). In summary, the top 10 hub genes obtained from the PPI network are closely related to tumorigenesis and tumor progression, which indicates that these hub genes might serve as prognostic markers and therapeutic targets for bladder cancer.

We also performed a module analysis on the constructed PPI network and selected the two most important modules, and we then performed a KEGG pathway analysis of the genes in these modules. The results showed that the genes in Module 1 are mainly enriched in the cell cycle and oocyte meiosis, and that the genes in Module 2 are mainly enriched in the complement and coagulation cascades, vascular smooth muscle contraction and FA. These results suggest that the development and metastasis of bladder cancer might be related to these pathways and that blocking the cell cycle and inhibiting adhesion signal transduction might be an effective treatment for bladder cancer.

## Conclusions

In summary, the purpose of this study was to improve our understanding of the molecular mechanisms underlying the development of bladder cancer through an integrated bioinformatics analysis that aimed to identify DEGs and the related pathways involved in bladder cancer progression. Our research also identified several key candidate genes and biological pathways that could aid the search for biomarkers and therapeutic targets of bladder cancer. However, further molecular biology experiments are required to validate the findings of this study.

## Supplemental Information

10.7717/peerj.6036/supp-1 Supplemental Information 1 The clinical information of the samples in each dataset.

10.7717/peerj.6036/supp-2 Supplemental Information 2 Integrated differentially expressed genes of bladder cancer.

## Additional Information and Declarations

### Data Availability

The following information was supplied regarding data availability: Gao, Xin (2018): Raw data and code.zip. figshare. Fileset. https://doi.org/10.6084/m9.figshare.7038296.v1.
