# Supplementary material for: Identification of key candidate genes and biological pathways in bladder cancer
Source: PeerJ. 2018 Dec 4;6:e6036. doi: 10.7717/peerj.6036 (PMC6284430; doi:10.7717/peerj.6036)
Supplement: Supplemental Information 2 [file peerj-06-6036-s002.docx]

| **DEGs** | **Gene names** |
| --- | --- |
| Upregulated | *TOP2A CDC20 WDR72 FCRLB UBE2C TCN1 PAFAH1B3 CRH NUSAP1 TYMS GJB2 CAMK2N1 CTSE EEF1A2 CDCA5 KRT8 TK1 PRC1 PRSS8 KRT7 TMEM54 CCNB2 LAD1 AURKA SDC1 CLIC3 HIST1H1C UHRF1 PTPRR MAPK13 KIF20A EVPL NCAPG KRT14 TPX2 TRIP13 SHMT2 SOX4 ISG15 MMP1 INA MIF NDUFA4L2 PTTG1 SPP1 TIMELESS ASPM PPP1R14B GJB6 PVRL4 SEMA3F TLCD1 CEP55 IGFBP3 PLA2G2F UBE2T KRT16 TTK FGFR3 JUP VEGFA HMGB3 GPT2 PPP4C SPAG4 CDH3 ANXA10 RAB25 CELSR3 OCIAD2 HMMR SCNN1G ANLN FSCN1 S100A9 ESM1 KRT20 CDT1 AURKB MCM4 PTK6 IGSF9 CDS1 MELK CA2 DTL SLC2A1 KIAA0101 CEACAM6 LMNB2 TM7SF2 KIF2C PBK FAM83H BUB1B PFKFB4 S100A14 SBK1 RANGAP1 CKS2 ASF1B CA9 CKAP2L LOC284837 CXorf57 SPAG5 CENPM MDK ERO1L SLC6A8 CCNB1* |
| Downregulated | *CNN1 PCP4 ACTC1 CFD FHL1 MYH11 CRYAB RGS2 FLNC PTGS1 TAGLN MFAP4 DES RASL12 HSPB6 SRPX PDLIM3 C2orf40 DPT CASQ2 SPARCL1 ACTG2 CYBRD1 FXYD6 SPON1 MRGPRF SLIT2 PTRF SELM TCEAL2 COX7A1 COL16A1 AEBP1 NR2F1 DPYSL2 APOD FGL2 PGM5 SDPR TMOD1 PALLD KIAA0367 RBPMS2 MYL9 SORBS1 MGP MSRB3 IGFBP6 SERPINA3 C7 CSRP1 COL6A2 KCNMB1 TSHZ3 OLFML3 ATP1A2 LMOD1 ITM2A C1S PPP1R14A FOXF1 FLNA CRISPLD2 CXCL12 TPM1 DPYSL3 FILIP1L P2RX1 GPX3 ALDH2 EDNRA REEP1 SMOC2 TIMP2 JAZF1 PTGIS STON1 CALD1 GHR GPR124 RGS1 PRKCDBP ZAK MYLK MAP1B PCOLCE2 PRICKLE2 CTGF JAM3 CKB CDH11 TUBB6 ALDH1A1 DCN SMTN GAS6 HSD17B6 SERPINF1 RARRES2 COL6A3 SERPING1 GYPC DDR2 CAV1 AXL EMP3 BIN1 FAM107A ITGA8 TMEM119 FBLN5 PLSCR4 NEXN INMT FBLN2 MYADM NDN EGR1 ITGA5 EPDR1 GAS1 LUM CLIP3 SCARA5 ACTA2 TCF4 ACOX2 OLFML1 LMCD1 PDK4 RRAS SFRP2 DUSP1 TGFB1I1 MAMDC2 FLJ21986 AOC3 WFDC1 ANTXR2 MXRA7 LGALS1 PAM PLAC9 TPM2 LHFP RGL1 COLEC12 ITPR1 RNF150 EFEMP1 COL15A1 SPARC RAMP1 SLC24A3 ADH1B COL5A1 TCF21 ABI3BP VIM CPXM2 KLF9 MFAP5 CYP1B1 ANG RNASE4 COL3A1 KLHL5 NFIA EMILIN1 CCND2 GSTM5 PELI2 DIXDC1 RPESP ADAMTS1 ACTN1 NXN ANXA5 CNN3 SETBP1 NFIB THBS2 ISLR DEGS1 SLC9A9 PEG3 JAM2 GNG11 PMP22 OLFM1 BNC2 PRUNE2 KLF2 FGF9 PNMA1 LIMS2 COL1A2 PPAP2B FAM43A SYNM LDB2 DACT3 SFRP1 KCTD12 ZEB2 MYOT TMEM158 PI16 SPRY1 MEF2C PLEKHO1 CYGB SGCE PROS1 GATA5 TCEAL3 PID1 NBEA DCHS1 A2M LAMB2 HLF ABCA8 HSPB2 CORO1C ZBTB20 SORBS2 EBF1 RHOJ ZBTB16 HTRA1 PTGDS* |

DEGs, differentially expressed genes
